# Simultaneous Detection of Dopamine and Serotonin—A Comparative Experimental and Theoretical Study of Neurotransmitter Interactions

**DOI:** 10.3390/bios9010003

**Published:** 2018-12-26

**Authors:** Felicia S. Manciu, Marian Manciu, John D. Ciubuc, Emma M. Sundin, Katia Ochoa, Michael Eastman, William G. Durrer, Jose Guerrero, Brayant Lopez, Mahendra Subedi, Kevin E. Bennet

**Affiliations:** 1 Department of Physics, University of Texas at El Paso, El Paso, TX 79968, USA; mmanciu@utep.edu (M.M.); jdciubuc@miners.utep.edu (J.D.C.); emsundin@miners.utep.edu (E.M.S.); kochoa2@miners.utep.edu (K.O.); wdurrer@utep.edu (W.G.D.); jaguerrero9@miners.utep.edu (J.G.); bmlopez3@miners.utep.edu (B.L.); msubendi@miners.utep.edu (M.S.); 2 Department of Biomedical Engineering, University of Texas at El Paso, El Paso, TX 79968, USA; 3 Border Biomedical Research Center, University of Texas at El Paso, El Paso, TX 79968, USA; 4 Department of Chemistry, University of Texas at El Paso, El Paso, TX 79968, USA; meastman@utep.edu; 5 Division of Engineering, Department of Neurologic Surgery, Mayo Clinic, Rochester, MN 55905, USA; Bennet.Kevin@mayo.edu; 6 Department of Neurologic Surgery, Mayo Clinic, Rochester, MN 55905, USA

**Keywords:** surface-enhanced Raman spectroscopy, neurotransmitters, dopamine, serotonin, computational analysis, simultaneous detection, label-free optical biosensors

## Abstract

With the goal of accurately detecting and quantifying the amounts of dopamine (DA) and serotonin (5-HT) in mixtures of these neurotransmitters without using any labelling, we present a detailed, comparative computational and Raman experimental study. Although discrimination between these two analytes is achievable in such mixtures for concentrations in the millimolar range, their accurate quantification remains unattainable. As shown for the first time in this work, the formation of a new composite resulting from their interactions with each other is the main reason for this lack of quantification. While this new hydrogen-bonded complex further complicates potential analyte discrimination and quantification at concentrations characteristic of physiological levels (i.e., nanomolar concentrations), it can also open new avenues for its use in drug delivery and pharmaceutical research. This remark is based not only on chemical interactions analyzed here from both theoretical and experimental approaches, but also on biological relationship, with consideration of both functional and neural proximity perspectives. Thus, this research constitutes an important contribution toward better understanding of neural processes, as well as toward possible future development of label-free biosensors.

## 1. Introduction

Neurotransmitters are chemical messengers that assist signaling between neurons. They are at the center of any primate’s behavior and psychomotor functions. Two of the most significant neurotransmitters present within the central nervous system are dopamine (DA) and serotonin (5-hydroxytryptamine or 5-HT). Dopamine belongs to the class of catecholamine neurotransmitters, acting as an excitatory neurotransmitter in nature, with serotonin acting as an inhibitory neurotransmitter. Dopamine, which is mainly synthesized in brain regions such as the substantia nigra pars compacta (SNpc), the ventral tegmental area (VTA), and the arcuate nucleus of the hypothalamus, has a significant influence on several pathways that include motor control, reward-based learning, arousal, addiction, activeness, motivation, and upper cognitive function [1,2,3,4,5,6,7,8,9,10]. Furthermore, depending on the postsynaptic neuron receptor type, DA can perform either as a fast neurotransmitter or as a slower neuromodulator in facilitating behavioral states [11]. Serotonin also has many functions that include psychomotor inhibition and regulation of emotions and mood, such as impulsivity, aggression, addiction, analgesia, eating disorders, cognition, and adaptation to stressors [12,13,14,15]. Two serotonergic pathways that originate from the dorsal (DRN) and medial raphe nuclei are known to innervate the cortical and subcortical structures through serotonergic release [16,17].

From pharmaceutical perspectives, there is a growing interest in these neurotransmitters, each of which is clinically effective on an individual basis. Independently, DA and 5-HT have been extensively studied and progress has been made in understanding their roles in neuronal systems [1,2,3,4,5,6,7,8,9,10,11,12,13,14,15,16,17]. However, much less has been reported in the literature regarding their intimate and complex relationship [18,19,20,21,22,23,24,25,26,27,28,29,30,31,32,33], especially from neurochemical perspectives [33,34]. Consequently, there is still a scientific need for understanding their interdependent chemical effects, and this has implications for the efficacy of adequate drug administration in the treatment of behavioral and mood disorder diseases. For example, from a neurobiological standpoint, in DA disorders such as Parkinson’s disease (PD), a monoamine neurotransmitter deficit develops into anhedonia and motivational loss, two strong indicators that are normally associated with depression [18]. Furthermore, the prevalence of depression in PD patients can reach over 50% in certain populations [19]. Dopamine-focused treatments, such as the dopamine precursor *Levodopa*, have been shown to lead to a reversal of depression symptoms and a noticeable improvement in the patient’s mood [20,21]. Monoamine pharmaceutical *Pramipexole* has also been demonstrated to serve as an effective antidepressant and mood stabilizer for patients with both bipolar depression and major depression disorders [22,23]. Thus, observations of the DA system and targeted treatments suggest that DA regulation does have an influence on the pathogenesis of behavioral and mood conditions, potentially in conjunction with 5-HT mechanisms and other catecholamine systems.

There is also a known opponency that occurs between DA and 5-HT, the latter serving as a strong modulator for all aspects of behavior, both regular and abnormal, especially concerning decision problems and conflicts that may arise [24,25,26]. However, despite the fact that DA and 5-HT have opposite functions, the two systems have a close relationship to each other, both functionally and proximally. As mentioned above, the dopaminergic system is primarily centered within the midbrain, with two of its primary pathways being the nigrostriatal pathway and the VTA projection [1,2,3,4,5,6,7,8,9,10,11]. Neurons arising from the SNpc connect to the striatum through the nigrostriatal pathway and perform as a motor behavior modulator. The VTA enters the mesolimbic and mesocortical pathways, affecting the limbic and cortical regions and is involved in behavioral regulation. The 5-HT pathway also originates in the midbrain, primarily in the dorsal and median raphe nuclei [16,17]. The DRN contains projections towards the cortex and striatal regions, with the median raphe nucleus entering the limbic regions. Therefore, considering the close proximity of serotonin and dopaminergic neurons, interplay is expected and observed between the two different classes of neurons. 5-HT’s inhibitory function affects dopaminergic neurons in both locations: the midbrain and the forebrain. Since the dorsal raphe neurons have a direct projection into the SNpc, 5-HT exhibits inhibitory regulatory effects on the DA neurons located in the substantia nigra. These effects can be observed when lesions are introduced intentionally, interrupting the dorsal raphe-nigra pathway and leading to systematic disruption of the inhibitory modulation of the DA network [27].

Aside from the natural 5-HT neuronal inhibition of DA neurons, ultrastructure analysis of the striatum reveals that the physical terminals of the 5-HT neurons are located in extremely close proximity to those of the DA neurons [28,29]. This is another indication that the two neural systems have the ability to interact with each other. Moreover, as 5-HT exhibits high concentrations in excretory vesicles with no extracellular enzyme to induce 5-HT degradation, the extracellular concentration of 5-HT is expected to be significantly large at such terminal locations [30,31]. DA transporters have been observed to exhibit 5-HT binding [29,31]. Similarly, 5-HT transporters have also been shown to bind DA [32], indicating not only a DA–5-HT transporter cross-interaction, but a potential for the neurotransmitters to interact as well. This is further evidenced by observations of DA and 5-HT co-release within the striatum [29].

From a molecular neurochemical viewpoint, both dopamine and serotonin contain structural features, such as hydroxyl groups, that make them good model compounds for studying intermolecular interactions among themselves, as well as with certain proteins [33,34,35]. However, while theoretical models were constructed and reported for such interactions [33,34,35], a direct correspondence between these models and experimental results through optical methods was not yet established at physiological levels. A major drawback here is that the majority of such analyses involve tagging analytes with a variety of fluorescent dyes or attaching them to different nanoparticles [36,37,38]. Labeling neurotransmitters does allow their detection by optical methods, but it also precludes monitoring their interactions with other analytes/neurotransmitters, and introduces changes to the overall chemistry and to the dynamic process. Thus, only through label-free and simultaneous detection can the desired computational and experimental comparison of neurotransmitter interactions be accurately evaluated. This is quite a challenging task, explaining the shortage of such reported results [39,40,41,42,43].

In this context, surface-enhanced Raman spectroscopy (SERS) is known to be a very sensitive method for label-free detection of any analyte at molecular levels. Consequently, it has been frequently applied for this purpose to different analytes, including neurotransmitters [40,41,42,43,44,45,46]. However, there is still no report of SERS use for simultaneous detection of multiple neurotransmitters at physiological levels. One reason for this is the fact that neurotransmitter vibrational lines exhibit shifts in frequencies and changes in intensities due to the chemicals’ interaction with the SERS metallic surfaces. Even complete disappearance of some vibrational lines has been reported, such as the DA vibrations at 750 and 795 cm^−1^ that were associated with either the in-plane phenolic ring bending mode or with the out-of-plane O–H and C–H bending modes [43,44,45,46]. Another more important reason arises from the similarity of the DA and 5-HT chemical structures, which induces a close overlapping of their dominant characteristic signatures in SERS measurements [40,42,43,44,45], complicating their accurate, simultaneous detection without labeling. For example, at very low concentrations, DA has SERS vibrational signatures around 1170, 1275, 1310, 1520, and 1620 cm^−1^ [43], whereas characteristic serotonin features are around 1140, 1235, 1350, and 1550 cm^−1^ [42]. Thus, considering at least ±5 cm^−1^ potential measurement errors in the frequencies of such vibrational lines, this will undermine accurate, simultaneous detection at physiological levels. At higher concentrations, where there is no need for SERS measurements, simultaneous detection is possible and has been reported [39]. Nonoverlapping signatures of 1290 cm^−1^ for DA and of 1550 cm^−1^ for serotonin were those that were primarily considered in order to distinguish between the neurotransmitters [39].

The proposed work here is to build on our previous simultaneous, label-free detection of dopamine and serotonin, with the challenge of accurately differentiating between them at physiological levels. To overcome the above limitation, theoretical analysis of the independent detection of DA and 5-HT, as well as of the detection of their potential interactions, will be discussed in detail and employed along with experimental findings in a comparative approach.

## 2. Experimental Procedure

### 2.1. Sample Preparation and Equipment

Dopamine (C_8_H_11_NO_2_, >99%) and serotonin (C_10_H_12_N_2_O, >99%) were purchased from Sigma Aldrich (Milwaukee, WI, USA) and used without further purification. Prior to the synthesis of 5-HT:DA, neurotransmitter mixtures with molar ratios of 7:3, 1:1, 2:3, 3:7, 1:4, and 1:9, 10^−2^ M concentrations of independent serotonin and dopamine in milli-Q water (of 18.2 MΩ∙cm resistivity at room temperature) were first achieved. The mixtures were vigorously shaken for a few minutes, then drop-cast on clean cover glass slips and vacuum dried to avoid unwanted neurotransmitter oxidation. The resulting thin films were stored under vacuum until further characterization.

To enable the detection of DA and 5-HT at nanomolar concentrations, the SERS methodology was employed. The process of synthesizing the silver nanoparticles (Ag NPs) that were used as SERS substrates for such measurements is described elsewhere [40,42,43]. The resulting Ag NPs colloidal suspension in ultrapure water was purified by centrifugation several times to remove the excess of organic and unreacted impurities before its mixing with solutions of combined neurotransmitters at different ratios. Concentrations of DA and 5-HT at 10^−7^ molar, which were obtained by successive dilution of each analyte in ultrapure water, were used to obtain mixtures with 4:1, 1:1, and 1:4 ratios. Next, 90 μL of the synthesized Ag NPs solution was mixed with 10 μL of each solution of the combined neurotransmitters. Finally, each resulting liquid sample bearing Ag NPs and a mixture of neurotransmitters was sonicated for 20 s, then drop-cast on clean cover glass slips, and vacuum dried. Again, the films were stored under vacuum until characterization.

The Raman measurements were acquired with an *alpha 300RAS WITec* confocal Raman system (WITec GmbH, Ulm, Germany). The 532 nm excitation of a frequency-doubled neodymium-doped yttrium-aluminum-garnet (Nd:YAG) laser, a 1024 × 127 pixel Peltier cooled CCD camera, and a 20X objective lens (Olympus, Tokyo, Japan) with a numerical aperture of 0.4 were used for data acquisition. The laser power output was kept at a few mW for Raman measurements of mixtures of neurotransmitters at 10^−2^ molar concentrations and was restricted to a much lower power output of about 100 μW for SERS measurements, to avoid sample damage. Multiple time series Raman spectra, each of 200 ms, were recorded in different locations of the samples and averaged. The *WITec Control 1.60* software was employed for this fast data acquisition. Background subtraction was also applied.

### 2.2. Computational Analysis

The *Gaussian-09* analytical suite software was employed for the quantum chemical density functional calculations. Prior to computing the Raman vibrational frequencies, energy optimization was performed. The Becke three hybrid exchange [47] and the Lee-Yang-Parr correlation functional, B3LYP [48], were used in these analyses. A 6-311++G(d,p) basis set was used for calculating the super-molecular form of the combined analytes. A LanL2DZ basis set, which takes into account the pseudopotentials for metal atoms, was employed for SERS simulations. Next, the *Gaussian-09* Raman output data were parsed using an in-house algorithm developed in C++ and subsequently converted to MATLAB version r2016a. A further conversion of Raman activities into relative Raman intensities following a previously reported procedure [49] was also performed. The value of the laser excitation (i.e., 532 nm = 18,796.99 cm^−1^) was used in this latter conversion. Finally, to assist with data plotting, all Raman peak intensities were normalized by a factor of *f* = 1e^−10^ and their shapes were modified by applying a Lorentzian band with a full width at half maximum (FWHM) of 7 cm^−1^.

## 3. Results and Discussion

**Figure 1:**
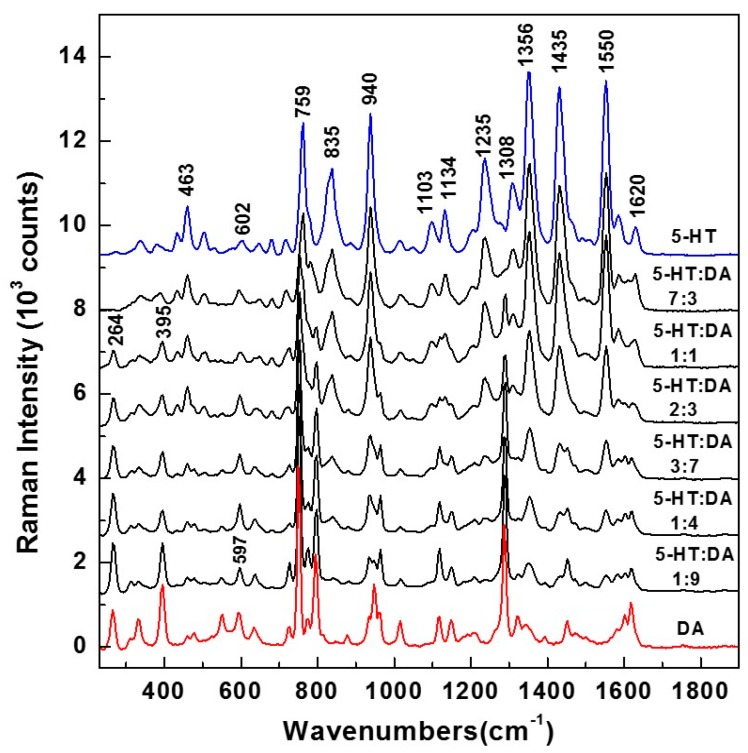
Raman spectra of serotonin (5-HT) and dopamine (DA) at 10^−2^ molar concentrations, for their mixtures at different ratios, as labeled. The spectra are vertically translated for easier visualization.

Although label-free detection of DA and 5-HT is reported in various works [39,40,41,42,43,44,45,46], only a single study suggests their potential chemical interaction with each other and the formation of a new compound [33]. This is surprising, considering their biological proximity and physiological co-existence within the striatum [28,29], as well as their chemical similarity. Thus, keeping in mind the likelihood of their interaction, as well as continuing our previous work on their simultaneous *in vitro* detection [39], we first explored a possible quantification of 5-HT and DA in different mixtures. It is known that Raman spectroscopy can provide such relative quantitative information. However, for an easier and more precise analysis, the high concentrations of 10^−2^ molar were first considered for such mixtures. The Raman results, of 5-HT and DA alone, and those of 5-HT:DA mixtures with ratios of 7:3, 1:1, 2:3, 3:7, 1:4, and 1:9, are presented in Figure 1. While the 5-HT Raman spectrum (blue color) has a multitude of characteristic vibrations at 463, 602, 759, 835, 940, 1103, 1134, 1235, 1308, 1356, 1435, and 1550 cm^−1^, sharp vibrational lines at 264, 395, 597, 750, 795, 935, 1013, 1117, 1148, 1290, 1450, and 1620 cm^−1^ can be observed in the DA Raman spectrum (red color).

**Figure 2:**
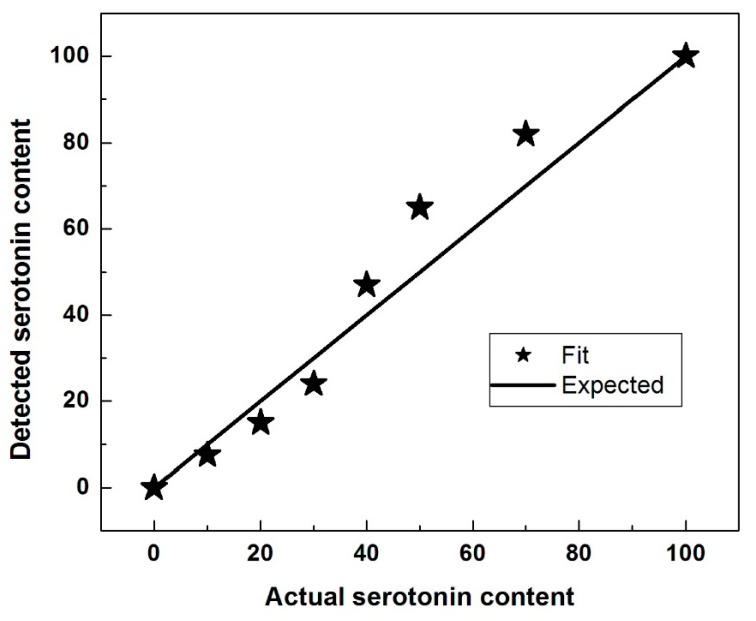
Statistical analysis of fraction of serotonin detected by fitting Raman measurements versus the actual fraction used in the sample preparation.

An expected decrease in the intensities of 5-HT Raman peaks are seen with less 5-HT. On the other hand, an increase in the intensities of DA lines, which correlates well with the increase in the amount of this analyte, can also be observed. These decreases/increases are more visible for the most intense vibrations, such as those at 835, 940, 1235, 1356, 1435, and 1550 cm^−1^ for serotonin, and at 750, 795, and 1290 cm^−1^ for DA. Besides close overlapping for the majority of Raman features, (i.e., around 602 cm^−1^ for 5-HT and 597 cm^−1^ for DA, 759 cm^−1^ for 5-HT and 750 cm^−1^ for DA, 940 cm^−1^ for 5-HT and 935 cm^−1^ for DA, 1103 cm^−1^ for 5-HT and 1117 cm^−1^ for DA, 1134 cm^−1^ for 5-HT and 1148 cm^−1^ for DA, and 1435 cm^−1^ for 5-HT and 1450 cm^−1^ for DA), there are also nonoverlapping vibrational lines at 835, 1235, 1308, and 1550 cm^−1^ for 5-HT and at 264, 395, 790, and 1290 cm^−1^ for DA). These latter vibrations can be used to distinguish between the two analytes. However, for better neurotransmitter identification and, more importantly, for more accurate quantification, all the Raman peaks were considered in the current analysis (i.e., not just the nonoverlapping vibrational lines), which is presented in Figure 2 and detailed below.

Under the assumption that the two compounds do not interact chemically, the Raman spectrum of a mixture of 5-HT and DA would be a linear superposition of the Raman spectra of the individual compounds. However, the data points indicated by stars for the samples of Figure 2 were obtained using the following procedure. Let actual fraction of serotonin with which a sample (mixture) was prepared be α_0_, so that the fraction of dopamine in that sample was 1-α_0_ (thus, by the horizontal axis scale of Figure 2, the samples have α_0_ values of 0, 0.10, 0.20, 0.30, 0.40, 0.50, 0.70, and 1.0). Using a variable parameter α, hypothetical linear superpositions of spectra were generated, each consisting of α times the experimental serotonin spectrum plus (1-α) times the experimental dopamine spectrum. For each mixture, a final value of α was obtained by fitting to minimize the sum of the squared differences between the data of the final hypothetical spectrum and the corresponding data of the spectrum that was obtained experimentally. Thus, the stars in Figure 2 represent points of the form (α_0_, α) where α is a final value from such a fit.

While from Figure 1, a reasonable qualitative estimation of the ratio of serotonin to dopamine can be obtained, the plotted α as a function of α_0_ presented in Figure 2 reveals that, quantitatively, this determination is not very accurate, particularly for comparable amounts of neurotransmitters in the mixture. This observation suggests the potential formation of a 1:1 compound, which can result from chemical interaction between a 5-HT molecule and a DA molecule. For example, if a quantity δ of this new 1:1 compound is formed in the mixture of α_0_ serotonin and (1−α_0_) dopamine, the actual ratio between 5-HT and DA should be $\frac{\propto_{0}-\delta }{1-\propto_{0}-\delta }$, which is smaller than $\frac{\propto_{0}}{1-\propto_{0}}$, if α_0_ < 0.5, and larger than $\frac{\propto_{0}}{1-\propto_{0}}$, if α_0_ > 0.5. Indeed, in Figure 2, our fitted value for α (i.e., detected serotonin content) systematically underestimates the serotonin at low 5-HT amounts in the mixtures and overestimates the serotonin at large amounts, substantiating the formation of this new compound.

**Figure 3:**
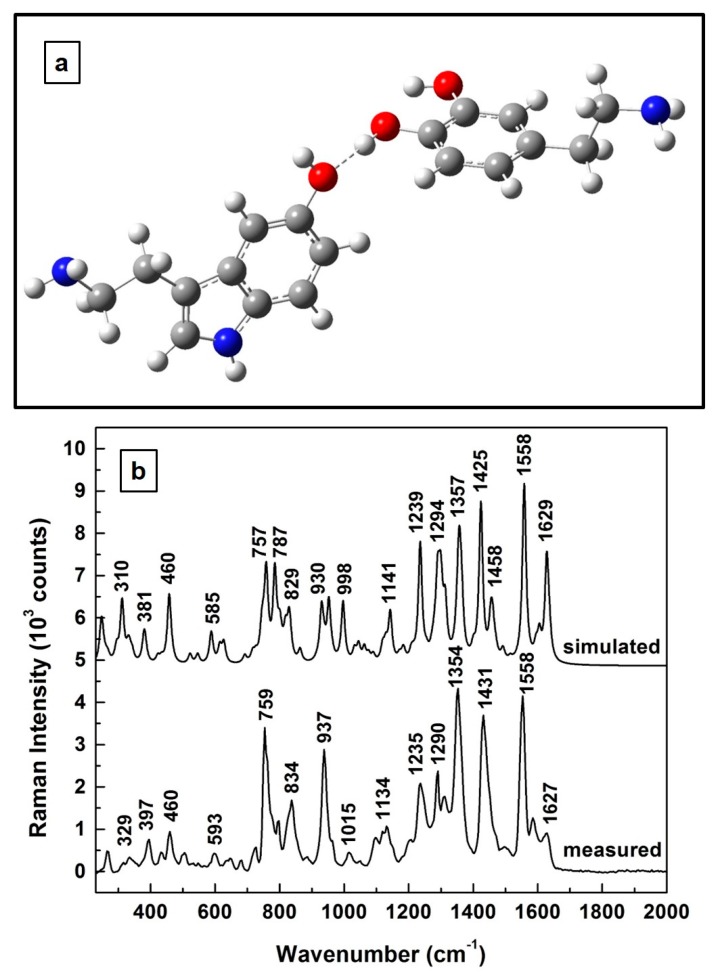
(**a**) Structural representation of serotonin—dopamine interaction and formation of a new 5-HT—DA complex through hydrogen bonding. Red and blue colors were used for oxygen and nitrogen atoms, respectively. (**b**) Theoretically calculated and experimentally measured Raman vibrations of 5-HT—DA composite. The spectra are vertically translated for easier visualization and appropriately labeled.

Consequently, it is natural to ask how the theoretically predicted Raman spectrum of the 1:1 molecular interaction of 5-HT and DA compares with that experimentally determined above in Figure 1. This evaluation is presented in Figure 3. As can be visualized in Figure 3a, where the predicted structural representation of this new composite is shown after appropriate energy minimization, a stable configuration occurs for a molecular interaction through hydrogen bonding between the two compounds. A serotonin molecule almost perpendicular to the dopamine benzene ring is also observed in this configuration. Furthermore, a look at the two associated Raman spectra, which are presented in Figure 3b, demonstrates good agreement between the computationally determined vibrations and the experimentally obtained ones, with differences of ±5 cm^−1^ for the positions of the most intense vibrational lines. A scaling factor of 0.98 has been used for the simulated frequencies to overcome the known systematic empirical errors originating from the force field constants employed in quantum mechanical approaches. Also, for easier visualization, a vertical translation of the spectra was performed. Thus, the similarity between these two Raman spectra further corroborates our assumption of the formation of a new composite.

**Figure 4:**
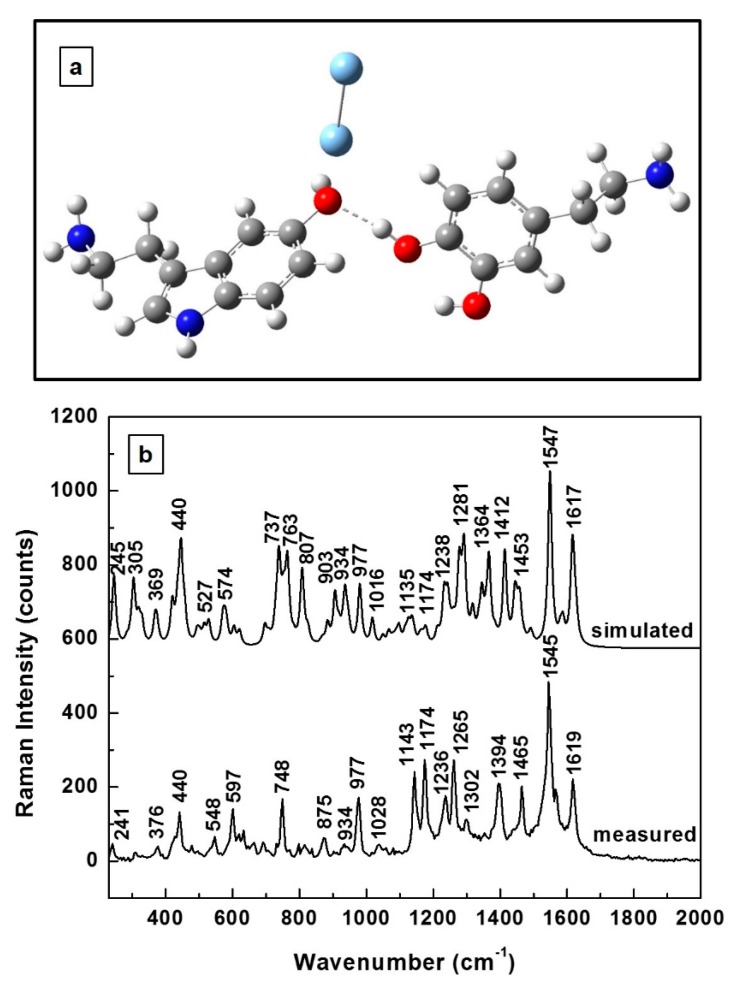
(**a**) Structural representation of new 5-HT—DA composite in the proximity of silver dimer after energy optimization. (**b**) Theoretically estimated and experimentally recorded Raman vibrational spectra of 5-HT—DA composite for a concentration of 10^−8^ M in the proximity of silver.

Since physiological levels of 5-HT and DA can be measured by employing SERS, we present in Figure 4a the energetically optimized molecular structure of this new 5-HT—DA hydrogen-bonded complex in the vicinity of the metallic substrate, which in this case is represented by the silver dimer. A different orientation of the serotonin molecule with respect to the dopamine molecule (namely, a slight rotation of the DA molecule) is observed in Figure 4a when compared to Figure 3a. Also observed in this figure is the silver dimer planarity with dopamine and its quasi-perpendicularity to serotonin, with a slight tilt towards the DA molecule. Again, relatively good agreement is seen in Figure 4b for the theoretically predicted and experimentally determined Raman spectra. A concentration of 10^−8^ M for the 5-HT—DA composite has been used for measurements in this case. A scaling factor of 0.965 was employed to adjust the simulated frequencies. Besides larger variances of ±8 cm^−1^ between the positions of some Raman peaks, an obvious difference in Figure 4b concerns the intensities of the features in the 740 cm^−1^–930 cm^−1^ region in comparison with those in the 1140 cm^−1^–1170 cm^−1^ region. In the simulated spectrum, most Raman lines have higher intensities than those experimentally obtained, while the opposite behavior is seen in the cases of the 1143 and 1174 cm^−1^ vibrations. Since these Raman peaks correspond to the ionic forms of DA and 5-HT [42,43], this observation suggests an abundance of ionized molecules in the vicinity of the SERS environment. Considering the fact that, at lower concentrations, the probability of neurotransmitters interacting is less than it is at their higher concentrations, the overall lower intensities of the Raman lines experimentally obtained are explained. However, despite this anticipated lower probability of 5-HT—DA composite formation at these lower concentrations, the experimentally measured abundance of ionized molecules deserves further analysis in the context of such a potential reaction.

The measured 748 cm^−1^ Raman feature in Figure 4b can be easily, but not correctly, associated with one of the strongest vibrations of the DA molecule at 750 cm^−1^. Since the same vibration corresponds to 5-HT, too [42], together with the fact that its presence was not detected in individual SERS measurements of DA at low concentrations [43,44,45], it is more likely that this feature belongs to the new 5-HT—DA biocomposite. This remark also highlights the importance and necessity of previous detailed investigations of an individual neurotransmitter for accurate identification of its characteristic vibrational lines in a SERS environment, as well as for comprehending its orientation in the proximity of the metallic surface. For example, for a similarly perpendicular orientation of the silver dimer with respect to that of the 5-HT molecule [42], a strong Raman peak at 895 cm^−1^ was previously measured [42] and is currently observed at 875 cm^−1^. Thus, although this feature might be attributed to the serotonin, its weakness in the current experimental data might also imply its association with the interaction of neurotransmitters with each other—again, with the new composite formation. However, its source is certainly that of serotonin, which is part of the 5-HT—DA biocomplex. Furthermore, other currently obtained vibrations, such as those at 440 and 977 cm^−1^, were also previously reported for a planar orientation of the silver dimer with respect to that of the dopaminequinone molecule (i.e., previously observed at 440 and 958 cm^−1^ [43]), suggesting the association of these features with the dopaminequinone form of DA. Moreover, since the strong intensity of the currently observed 1547 cm^−1^ Raman peak is more like that of the 1527 cm^−1^ line of the DA anion [43], again for its similar planar orientation in the proximity of silver dimer, this ionic form of DA is the main contributor to this line. Thus, while all the currently observed Raman features can be associated with the analytes themselves, in their different forms, considering the large shifts in their frequencies of ± 20 cm^−1^, their association with the existence of a new compound cannot be excluded, either.

**Figure 5:**
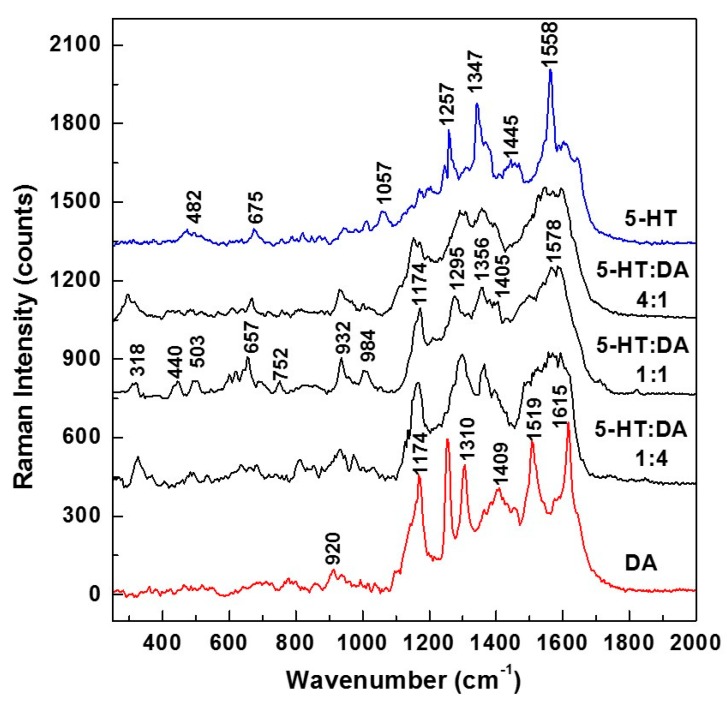
Overall averages of 140 Raman spectra recorded in different spots on SERS mixture samples with different ratios, as labeled (seven different time series acquisitions, of 20 spectra each and at 200 ms per spectrum). The individual Raman spectra of 5-HT and DA are also presented for comparison.

For better comprehension of the likely formation of the 5-HT—DA composite and discrimination between 5-HT and DA at these low concentrations of about 10^−9^ molar, we present in Figure 5 the overall averages of 140 SERS spectra (seven different time series acquisitions of 20 SERS spectra each, at 200 ms per spectrum). These data were collected in different locations on samples that were prepared with different 5-HT:DA ratios, namely 4:1, 1:1, and 1:4. Again, for an easier comparison of these SERS average mixture spectra with those of 5-HT and DA, we also present in Figure 5 the SERS spectra of each neurotransmitter (blue line for serotonin and red line for dopamine).

A glance at these spectra reveals that at concentrations close to physiological levels, even in the spectra of the standard neurotransmitters, vibrations associated with their ionic forms can be seen. This affirmation is based on the weak Raman lines at 482, 675, and 1057 cm^−1^ for 5-HT, and on the strong peak at 1174 cm^−1^ for DA. These ionic forms are expected during sample preparation (when the neurotransmitters get dissolved in water). More interesting is the observed trend of decreasing intensity of the 1174 cm^−1^ vibrational line with serotonin addition. This observation, besides further supporting the concept of 5-HT—DA formation as the DA and 5-HT ionic forms combine with each other, also confirms that, indeed, serotonin has an antioxidant effect on dopamine. The deprotonation of DA and its transformation into the dopaminequinone form [43] is now compensated by the addition of serotonin and the resulting molecular hydrogen sharing between these two analytes. The likely existence of the 5-HT—DA compound is also evident in these SERS Raman spectra through the appearance of other weak features at 440, 752, and 984 cm^−1^. The weakness of these peaks also corroborates our previous assumption of a lower probability for the formation of this new compound at concentrations characteristic of physiological levels. Not only does the potential existence of this new hydrogen-bonded complex further complicate the analysis, but the observed broadness of the features in the 1200–1600 frequency region, as well as their close overlap, makes the discrimination between and accurate quantification of these analytes a very challenging task.

## 4. Conclusions

In this research, we take on the challenge of detecting and quantifying the amounts of 5-HT and DA in mixtures of these neurotransmitters at concentrations characteristic of physiological levels and without using any labelling. For achieving a better understanding of whether such a task can be completed, at least with an *in vitro* chemical approach, as well as building on our previous work on these neurotransmitters’ simultaneous *in vitro* detection [39], a comparative computational and Raman experimental study is presented and comprehensively discussed here. Even though, from a biological perspective, the detection of these neurotransmitters is expected to be much more complex, their biological proximity and physiological co-existence within the striatum [28,29], as well as their known interrelated biofunctionality, are not only intriguing, but also suggest their potential interaction. This interaction is indeed confirmed in this study and more evident in their mixtures at higher concentrations, in the millimolar range. Such higher neurotransmitter concentrations of 10^−2^ molar were first considered to better evaluate their potential for quantification. As indicated in the current statistical analysis (see Figure 2), their interaction occurs for a 1:1 compound (i.e., 5-HT:DA ratio of one). This remark can be explained considering that the concentration of the 1:1 compound (via the association/dissociation equilibrium) is proportional to the product of concentrations of the DA and 5-HT constituents. Therefore, it is negligible when one constituent has much lower concentration than the other and it is largest at an equal mixture of constituents, as Figure 2 indicates.

Based on this observation, we further compared the theoretically predicted Raman vibrations of this 5-HT—DA composite with those experimentally obtained. A hydrogen sharing bond between the neurotransmitters was assumed for their interaction. The relatively good agreement between these two Raman spectra at higher neurotransmitter concentrations (except for ±5 cm^−1^ difference in the positions of the most intense vibrational lines), reassures us of the formation of this biocomplex. Since detection of neurotransmitters at physiological levels is achievable only by employing the SERS method, we also engaged in this more demanding investigation, again from both perspectives, theoretical and experimental. Although a good agreement between the two Raman spectra (i.e., simulated and measured) was still obtained, to eliminate potential discrepancies between the experimentally determined data, as well as to more accurately identify the observed vibrational lines, additional analysis was also performed. For such investigations, averages of 140 SERS spectra that were recorded in different sample locations, and for samples prepared with 4:1, 1:1, and 1:4 ratios, were considered.

While the formation of this new 5-HT—DA composite is confirmed by these data, too, its presence further obscures the potential discrimination between and individual quantification of 5-HT and DA in mixtures of these analytes. Also, the broadness and weakness of the observed Raman lines additionally complicate this analysis. In conclusion, although quantification of the two neurotransmitters cannot yet be accurately done and more research should be performed, the finding of the potential formation of this new compound might in itself open new avenues for its use in drug delivery and pharmaceutical research. Again, more research should be done to completely define its chemical structure and its biological implications. However, such research is beyond the scope of the research presented here.
